# A systematic review on the assessment of cerebral autoregulation in patients with Large Vessel Occlusion

**DOI:** 10.3389/fneur.2023.1287873

**Published:** 2023-11-17

**Authors:** Faheem G. Sheriff, Arghal Ahmad, Mehmet E. Inam, Rakesh Khatri, Alberto Maud, Gustavo J. Rodriguez

**Affiliations:** ^1^Department of Neurology, Section of Interventional Neurology, Texas Tech University Health Sciences Center El Paso, El Paso, TX, United States; ^2^Ziauddin University, Karachi, Sindh, Pakistan; ^3^University of Texas Health Science Center at Houston, Houston, TX, United States

**Keywords:** cerebral autoregulation, large vessel occlusion, acute ischemic stroke, mechanical thrombectomy, cerebral blood flow

## Abstract

As the majority of large vessel occlusion (LVO) patients are not treated with revascularization therapies or efficiently revascularized, complementary management strategies are needed. In this article we explore the importance of cerebral autoregulation (CA) assessment in the prediction and/or modification of infarct growth and hemorrhagic transformation. In patients with LVO, these are important factors that affect prognosis. A systematic search of the PubMed, EMBASE databases and a targeted Google search was conducted, resulting in the inclusion of 34 relevant articles. There is an agreement that CA is impaired in patients with LVO; several factors have been identified such as time course, revascularization status, laterality, disease subtype and location, some of which may be potentially modifiable and affect outcomes. The personalized CA assessment of these patients suggests potential for better understanding of the inter-individual variability. Further research is needed for the development of more accurate, noninvasive techniques for continuous monitoring and personalized thresholds for CA.

## Introduction

**Figure 1:**
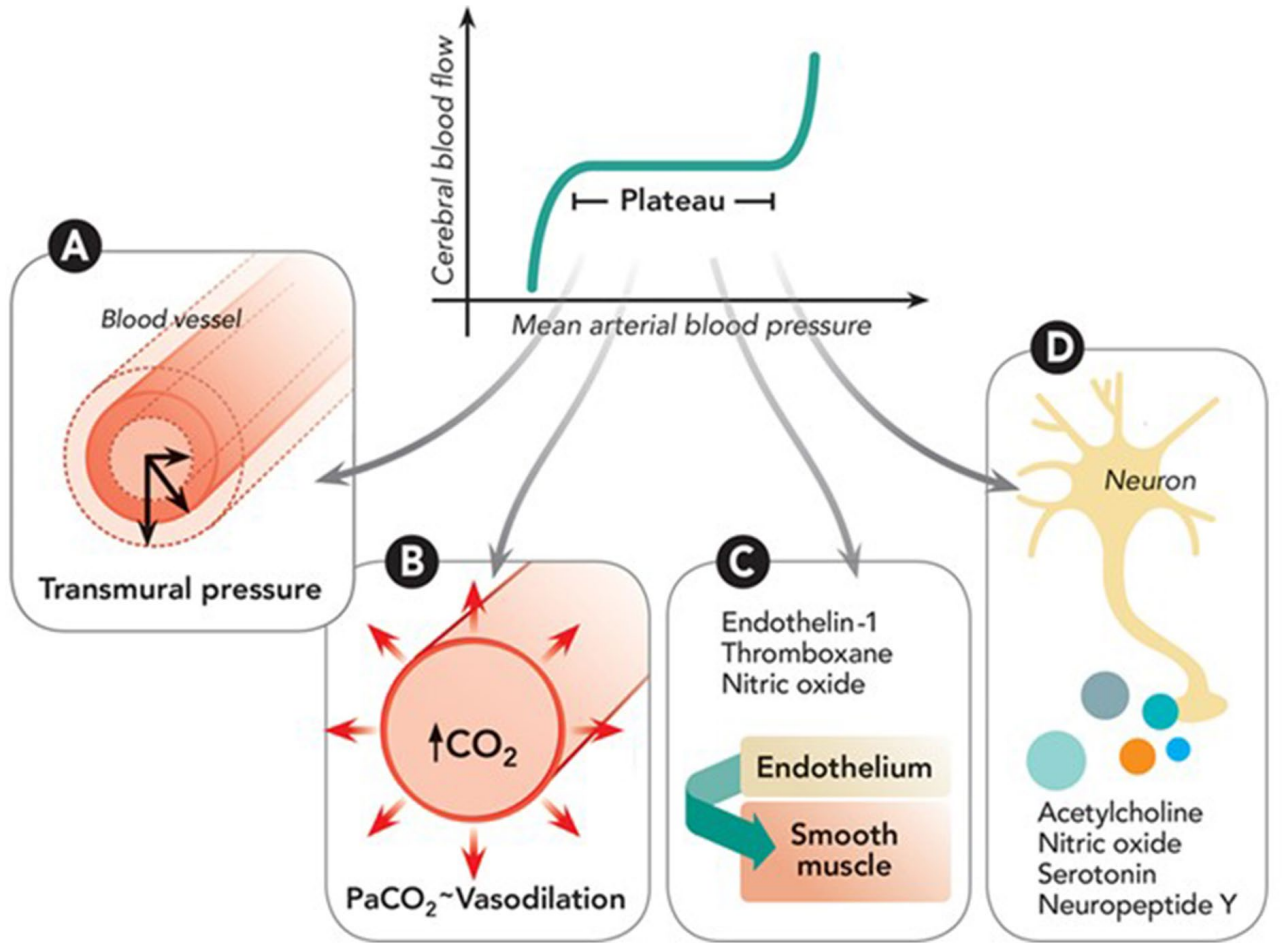
Depiction of cerebral autoregulation processes: **(A)** Myogenic response adjusts arterial size based on transmural pressure via muscle activity. **(B)** Metabolic response modulates small artery size based on carbon dioxide (CO₂) levels from oxidative phosphorylation. **(C)** Endothelial response involves secretion of agents like nitric oxide and endothelin-1 that act on smooth muscle. **(D)** Neurogenic response involves neuroglial cells regulating vessel diameter through various vasoactive neurotransmitters. Reproduced with permission from the publisher Walters Kluwer Health, Inc., for the original article by Rivera-Lara et al. (2).

Cerebral Autoregulation (CA) is the intrinsic capacity of the vasculature in the brain to maintain constant blood flow despite variations in systemic blood pressure (1). This phenomenon is mediated by myogenic and neurogenic processes (via neurotransmitters) and driven by changes in cerebral metabolism, partial pressure of oxygen (PaO_2_), or carbon dioxide (PaCO_2_), temperature, and intracranial pressure among others (See Figure 1). There is impaired CA in all types of acute ischemic stroke (AIS) patients, and it likely leads to worse clinical outcomes. Failure of CA is associated with secondary brain injury that may occur as an extension of the initial ischemic core, “no-reflow phenomenon” (3), cerebral edema, or hemorrhagic transformation.

Large vessel occlusion (LVO) accounts for approximately 30 to 40% of all acute ischemic stroke (AIS) cases. Currently, only 10–20% of patients with LVO undergo endovascular treatment. Out of those patients that receive endovascular treatment, effective recanalization is achieved in about 70% of the cases, yet only approximately 46% will have an independent outcome at 90 days (4). The importance of optimizing CA in these patients during different phases of AIS including during intensive care has not yet been well established.

Optimization of CA in LVO patients could potentially have a significant impact on their clinical outcome. We hypothesize that patients that are most likely to benefit include those that are awaiting to undergo endovascular treatment, those that were treated but did not have effective recanalization and those patients that lack adequate arterial collaterals. While there are reviews about CA in the general acute ischemic stroke population, there are no specific comprehensive in-depth analyses and/or literature reviews regarding CA in patients with LVO.

## Methods

PubMed and EMBASE databases were searched on October 06, 2022, and October 11, 2022, respectively. Stroke, Brain or Cerebrum or Cerebrovascular Circulation, Homeostasis, Procedures or Thrombectomy or Mechanical Thrombolysis or Thrombolytic Therapy or Tissue Plasminogen Activator were selected as relevant Medical Subject Headings (MeSH) and combined using the ‘AND’ clause. All MeSH terms were exploded. All keywords from the PubMed MeSH library were included along with associated MeSH terms using the ‘OR’ clause, except for expired keywords or keywords that had a comma within the term. Additional field-appropriate keywords were added to the final search to expand the query, specifically “large vessel occlusion” and its variants, “acute ischemic stroke” and its variants, “dysautoregulation,” and “cerebral.” The same search was conducted on EMBASE with associated EMTREE terms. All keywords were matched to their synonyms and additional synonyms were included when identified on EMBASE database. The final searches are included as supplemental document (Appendix I). We selected only original research articles written in the English language and excluded non-human subject research, case reports, case series of fewer than five subjects, abstracts, book chapters, studies that did not measure CA, and reviews including commentaries, editorials, short surveys, and letters. Additional targeted Google search was conducted based on topics identified through the primary search from PubMed and EMBASE. Specifically, we identified recent papers studying the effects of perioperative BP management, anesthesia type during endovascular thrombectomy, and PaCO2/ETCO2 levels on *CA.*

## Results

**Figure 2:**
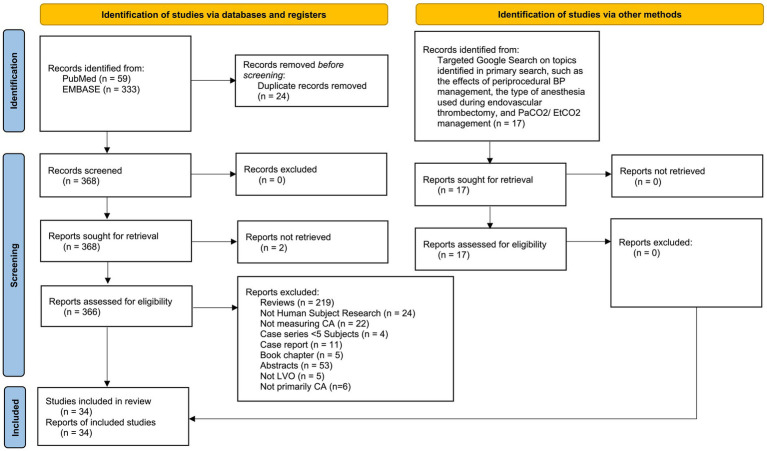
PRISMA diagram.

The PubMed and EMBASE search strategy yielded 368 studies after duplicates were removed. Of these, 366 articles were accessible and assessed for eligibility, while two could not be retrieved. Among the accessible articles, 17 met the inclusion and exclusion criteria. In addition, the targeted Google search added 17 more qualifying articles, resulting in a total of 34 articles for our review (Figure 2). We reported the results in a discussion format under different relevant subheadings.

### Cerebral autoregulation monitoring

An important obstacle in the bedside monitoring of CA is the lack of a reliable and reproducible method, a gold standard. It is recommended that for appropriate CA assessment, Cerebral blood flow (CBF), systemic blood pressure (SBP) and partial pressure of CO_2_ (PaCO2) or a surrogate should be recorded, as the latter is a strong factor affecting CBF. Researchers have devised a handful of methods to assess changes in CA; these include static and dynamic methods (1). Cerebral autoregulation can be evaluated by measuring relative CBF changes in response to a steady-state change in the SBP (static method) or during the response to a rapid spontaneous change in SBP (dynamic method). Xenon CT, MRI, and PET scans are examples of static modes of obtaining CA metrics, but they are limited in their utility as they only provide a “snapshot” overview. Therefore, not only are they not optimal for continuous monitoring but also may require pharmacological induction with potential undesired effects.

Dynamic methods are either based on *time* or *frequency* analysis and utilize surrogates of CBF such as transcranial Doppler (TCD), laser Doppler flowmetry, diffuse correlation spectroscopy (DCS) and near-infrared spectroscopy (NIRS) (1) to assess the relation between CBF and SBP changes. Various linear and non-linear mathematical techniques are used to analyze this relationship. Examples of techniques utilized in the *time* domain include cerebrovascular resistance (CVR), cerebrovascular resistance index (CVRi), pulsatility index (PI), critical closing pressure (CrCP), rate of recovery (RoR), and autoregulatory index (ARI). See Table 1 reproduced from van Beek et al. (5).

**Table 1:** Overview of various dynamic quantifications of cerebral autoregulation in the time domain [reproduced with permission from Van Beek et al. (5)].

CA measurement	Abbreviation	Unit/value	Definition
Cerebrovascular resistance	CVR	mmHg per mL per min	$\frac{BP_{\mathrm{mean}}}{CBF_{\mathrm{mean}}}$
Cerebrovascular resistance index	CVRi	mmHg per cm per sec	$\frac{BP_{\mathrm{mean}}}{CBFV_{\mathrm{mean}}}$
Pulsatility index	PI	–	$\frac{CBFV_{sys}-CBFV_{\mathrm{dias}}}{CBFV_{\mathrm{mean}}}$
Critical closing pressure	CrCP	mmHg	The extrapolated value of BP at which CBF approaches zero
Rate of recovery	RoR	CBFV per sec	$\frac{\mathrm{CVRi}/T}{BP}$
Autoregulatory index	ARI	0–9	Nine models for CA: 0 indicates no autoregulation 9 indicates very fast regulation
Correlation coefficient	Mx	0–1	Pearson’s correlation coefficient between BP and CBFV

BP, blood pressure; CBFV, cerebral blood flow velocity.

**Figure 3:**
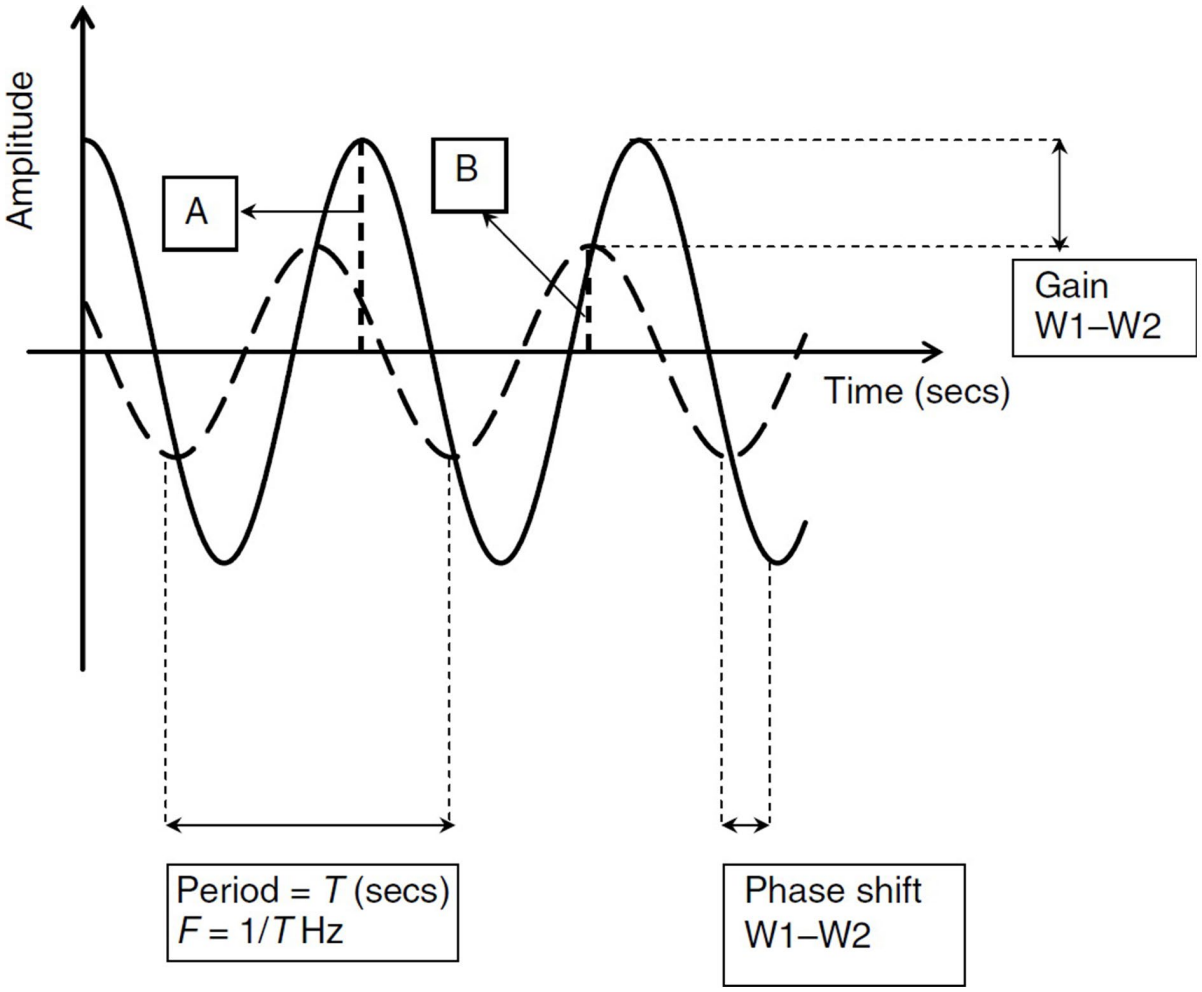
Analysis of Transfer Functions: This figure provides a visual interpretation of transfer function analysis using two sinusoidal waves with identical periods (T). The frequency (F) of these waveforms is determined as 1/T. **(A)** Illustrates Waveform 1 (W1), shown as a solid line, with its amplitude. **(B)** Depicts Waveform 2 (W2), indicated by a dashed line, and its amplitude. The concept of gain in this context refers to the reduction effect observed from W1 to W2, while the phase shift indicates the time lag between W1 and W2. In this scenario, W1 serves as the input and W2 as the output in the transfer function. The system’s controller reduces the amplitude of the input, resulting in a diminished amplitude (gain) in W2. The temporal displacement of W2 compared to W1 is depicted through the phase shift. Reproduced with permission from the publisher SAGE Publications for the original article by van Beek et al. (5).

In the *frequency* domain analysis, the regulation of CBF in response to changes in SBP can be described using transfer function analysis (TFA) which includes the following parameters:

- Gain, which is the damping effect between input and output of the transfer function (SBP and CBF, respectively); it depicts the attenuation of cerebral blood flow velocity (CBFV) amplitude for every unit change of SBP.
- Phase shift, which is the time delay in transmission of SBP to CBF and is usually measured in degree radians.
- Coherence, which is the assessment of linearity of the relation between input and output, i.e., SBP and CBFV fluctuations, respectively. See Figure 3 reproduced from van Beek et al. (5).

While there is no universally accepted method to monitor CA, there are unique advantages and disadvantages for each modality. For instance; while TCD has good temporal resolution and provides an indicator of macrovascular autoregulation, it requires adequate temporal acoustic windows in the patient, which are lacking in 5 to 37% of the population (6, 7). In addition, the technique is highly operator-dependent. Near infrared spectroscopy (NIRS) is one common alternative to TCD; it utilizes near infrared light to estimate the relative oxyhemoglobin and deoxyhemoglobin concentrations in the cortical surface to provide an indirect marker of CBF (1). It reflects the state of autoregulation at the microvascular level and therefore serves as an indicator for superficial cortical perfusion pertaining to the vascular territory being assessed (1). Therefore, it has good temporal resolution but lacks spatial resolution. Another optical modality which utilizes scatter of red blood cells to provide a direct estimate of CBF is diffuse correlation spectroscopy (DCS) (8). This technology shares the non-invasiveness, deep tissue penetration, excellent time-resolution, and potential for continuous bedside monitoring with NIRS but was designed to overcome some of its limitations by providing a more direct and distinct measure of CBF (9). The combination of the two techniques provides a more comprehensive assessment of cerebrovascular hemodynamics, autoregulation and cerebral metabolic rate of oxygen extraction (CMR02); this combination is referred to as hybrid diffuse optical technologies (10).

The most suitable modality is therefore likely dependent on the specific condition being studied, patient specific factors and presence of other intrinsic limitations. In our opinion, two or more complementary modalities may be necessary to adequately assess CA in LVO cases.

### Time course of impaired CA in AIS

Among AIS patients of different subtypes, Salinet et al. (11) demonstrated significantly lower cerebral blood flow velocity (CBFV) in the affected hemisphere within 72 h compared to a historical control, however the sample of patients studied was not limited to LVO patients (11). The CA index was mainly impaired at 2 weeks but returned to baseline at 3 months during the clinical recovery (11). In a more recent study, which included confirmed LVO patients, the CA was assessed at multiple time points (1–5 days) from symptom onset. Utilizing TCD and TFA, it was observed that CA was initially impaired and eventually a trend toward normalization was demonstrated at approximately 3–4 days (12). The degree of impairment and the rate of recovery was variable and contingent on the degree of revascularization.

In summary, impairment of CA has been documented in the acute phase (<48 h), with changes in phase shift in the ipsilateral hemisphere of subjects suffering from LVO. This gradually normalizes over time from the sub-acute (48 h-7 days) to the chronic phase when measured at 3 months (12, 13).

### Laterality of impaired CA

Impaired CA is usually documented in the affected hemisphere compared to the contralateral unaffected hemisphere. In a study by Petersen et al. (14) involving LVO patients, the TFA phase shift was lower (correlating with impaired autoregulation) in the affected hemisphere compared with the unaffected hemisphere at 48 h. Interestingly, in another study measuring laser speckle imaging and MAP in patients undergoing decompressive craniectomy for malignant stroke, the degree of CA impairment was significantly greater in critically hypo perfused viable tissue (perfusion 20 to 40%) compared to the non-infarcted (perfusion 100%) or infarcted tissue (perfusion <20%) (15). According to the INFOMATAS project; dynamic CA status should be evaluated in the acute phase (under 48 h) in the affected hemisphere in LVO and /or severe stroke particularly in context of recanalization therapies (16); which should then be correlated with short and long term clinical outcomes – the grade of recommendation was “strong.” The same group of authors recommend assessment of CA in the unaffected hemisphere during the acute phase which can yield “useful” information (moderate grade) (16).

While it is worth mentioning that in LVO stroke patients CA has been noted to be impaired in the affected hemisphere consistently, interestingly the contralateral side may also show CA impairment even after recanalization of the affected side. Several mechanisms have been postulated but the exact mechanism remains unclear (17–19).

### Posterior circulation CA

Several processes affecting mainly the posterior fossa have been linked to an inadequate CA; posterior reversible encephalopathy syndrome, migraine, hypertensive crises and even hypertensive intracerebral hemorrhage (20–22). The CA of the posterior circulation stemming from the vertebral arteries has not been well investigated. An earlier study in healthy human subjects suggested that perceived differences between CA in the anterior and posterior circulation were likely a representation of metabolic vasodilation related to visual cortex activation and not necessarily an inherent difference in the autoregulatory characteristics of the posterior circulation. However, the study used TCD and end tidal CO2 and appeared to test neurovascular coupling which is more and more recognized as an independent mechanism from that of CA. Although, the posterior circulation is believed to have a precarious or absent sympathetic innervation compared to the anterior circulation, an alteration in neurovascular coupling may not necessarily mean an alteration in CA (23).

In patients with severe basilar stenosis, it has been observed that when TFA phase-shift was decreased suggesting impaired CA, patients were more likely to suffer AIS with a poor 90-day functional outcome (24). Another interesting observation is that when CA was assessed in patients with pre-eclampsia using TFA phase shift, impairment was noted in the middle cerebral arteries, however given the MRI brain abnormalities noted in the posterior fossa, the authors assumed a worse CA impairment in the posterior circulation (20).

### Extracranial atherosclerotic disease (anterior circulation)

Extracranial atherosclerosis such as internal carotid artery (ICA) stenosis has been well studied and been found to cause impaired CA (25–29). This failure in autoregulation is ipsilateral to the stenotic vessel and is not only a result of severe ICA stenosis (70–99%) but it has also been seen with moderate stenosis (50–69%) and can be effectively restored with revascularization (30). Many studies deducing CA in various types of stroke fail to take into account the effects of extracranial stenosis as it is a known vascular risk factor for AIS (31).

### Intracranial atherosclerotic disease (ICAD)

The most common cause of AIS in the Asian, Hispanic and African population is ICAD. Although the most common presentation of ICAD is with a transient ischemic stroke or small stroke, occasionally it can present with LVO which is most likely associated with a different autoregulatory profile. While data in the setting of LVO in ICAD patients is scant, studies performed in ICAD patients in the context of secondary prevention suggest impaired *CA.*

ICAD has three well-described mechanisms of causing AIS: perforator artery atherothrombosis, artery-to-artery embolism and hypoperfusion; the latter mechanism is most likely to respond to endovascular therapies but also more susceptible to harm if there is collateral failure. Corroborating this view, one case control study demonstrated that in those ICAD patients with stroke related to hypoperfusion, ipsilateral CA was closely correlated with collateral status and CBF as assessed by MRI ASL (arterial spin labeling) (32). The same study showed unilateral CA impairment with perforator artery atherothrombosis, on the contrary bilateral impairment was seen with other mechanisms.

In general, studies assessing CA in ICAD are scant (33). Interestingly in asymptomatic middle cerebral artery (MCA) stenosis, CA has been demonstrated to be impaired unilaterally while symptomatic MCA stenosis was found to affect CA bilaterally (34). The degree of stenosis was directly related to CA impairment (35, 36).

### Moyamoya disease

Moyamoya disease (MMD) is a rare cerebrovascular entity with progressive stenosis of the distal internal carotid arteries at the level of the ICA terminus and their proximal branches. It is more commonly seen in pediatric patients. While LVO is an uncommon presentation of MMD, there have been reports of patients requiring IV thrombolysis and/or thrombectomy (37). An earlier study demonstrated that dynamic CA was impaired in the early stages of MMD and that autoregulatory parameters are well correlated with angiographic stages of MMD (38). Indirect or direct revascularization procedures are often necessary and have variable success rates. Utilizing NIRS, one study was able to identify the lower limits of autoregulation (usually 55–65 mmHg) and optimum MAP goals (ranging from 70–90 mmHg) for pediatric patients undergoing indirect revascularization (39). Another study utilizing cerebral oximetry index (COx) and coherence from transfer function analysis (TFA) was able to predict postoperative infarction (40).

### Modifiable factors affecting CA in AIS

#### Effect of revascularization status on CA

##### Intravenous (IV) thrombolytics

Agents such as tissue plasminogen activator (tPA) and Tenecteplase (TNK) have been the mainstay treatments of AIS in the early time window (41). A study by Nogueira et al. (42) using the Autoregulation index (ARI) as a surrogate for CA measured by TCD demonstrated that CA was impaired in subjects who did not respond to IV thrombolysis and had worse outcomes as assessed by NIHSS scores at 24–48 h. This was corroborated by another study which demonstrated increasingly impaired CA especially in the affected hemisphere over the first 5 days after unsuccessful IV thrombolysis (43).

##### Mechanical thrombectomy (MT)

When used with or without preceding IV thrombolysis, MT is now the standard of care for LVO patients presenting within the first 6 h and in select cases up to 24 h. Despite successful recanalization, some patients do not have favorable outcomes (12, 44). This might be due to reperfusion injury (45) or the ‘no-reflow phenomenon’ (3), among other factors. A multi-center study assessing dynamic CA patients after MT, showed that subjects with complete (Thrombolysis in Cerebral Infarction (TICI) 3) recanalization were associated with a more favorable autoregulation profile compared to incomplete (TICI 2b or worse) recanalization (12).

#### Effect of blood pressure control

##### Fixed targets

It has been shown that the severity of CA impairment in AIS correlates with blood pressure variations (14). A target below 180/105 mmHg in the first 24 h, during and post MT has been recommended in the American Heart Association/American Stroke Association (AHA/ASA) guidelines as an extrapolation from IV thrombolysis literature (46, 47). Previously published observational studies had shown a relationship between systemic hypertension, hemorrhagic transformation and poor outcomes post MT (48, 49). The BEST study, a large multicenter prospective study demonstrated in unadjusted analysis that a peak SBP post MT of 158 mmHg was able to discriminate good from poor functional outcomes (with higher SBP portending worse outcomes) (50, 51). The ENCHANTED 2/MT was a multicenter blinded-endpoint randomized trial which enrolled 821 patients into an intensive and a less intensive arm for BP goals post MT. The intensive arm with a BP goal <120 mmHg had a higher percentage of subjects with neurologic deterioration and mRS score(3–5) at 90 days (*p* < 0.001) than the less intensive treatment cohort with BP targets 140–180 mmHg (50, 52). There was no difference in rates of symptomatic ICH. The recently published BEST 2 study, demonstrated that SBP targets after endovascular therapy of less than 140 mmHg and less than 160 mmHg, did not demonstrate significantly lower infarct volumes. While it did not meet prespecified criteria for futility compared with a target of 180 mmHg or less, there was low probability of lower SBP targets demonstrating benefit in future clinical trials (53). These results highlight the limitations of fixed BP targets.

##### Personalized targets

Tailored blood pressure goals to the state of CA in an individual patient at a given point in time is an attractive alternative to fixed BP goal in light of the afore described RCT data. Petersen et al. (14) studied one such personalized approach post MT in patients with AIS. The authors studied different thresholds; predetermined or fixed according to the guidelines and thresholds based on perfusion status. For personalized targets, using NIRS and time-correlation analysis they deduced the mean arterial pressure (MAP) at which CA is most preserved (MAP opt). Time spent above the upper limit of autoregulation (ULA) and below the lower limit of autoregulation (LLA) was calculated for both groups; 30% of the subjects had favorable outcomes (mRS 0–2) at 3 months (14). The study showed that more time spent within personalized AL (79%) was associated with a good outcome, less hemorrhagic transformation and infarct growth compared to those with poor outcomes who spent 60% within personalized AL. Every 10% increase in time spent above ULA was associated with a 1.9-fold increase in the odds of shifting toward a worse outcome on the mRS at 90 days (14).

#### Effect of anesthesia type

General Anesthesia (GA) has traditionally been used for some neurointerventional procedures over conscious sedation (CS) as it lowers the risk of procedural injury, facilitates blood pressure control, protects the airway, and makes the procedure more tolerable for patients. Potential drawbacks include delays to treatment, drops in MAP/CPP and the effect of general anesthetic agents on *CA.* Earlier retrospective studies and meta-analyses showed that subjects exposed to GA had poorer outcomes compared to CS (54–57). These studies had certain limitations including being retrospective and not taking into account the type of anesthetic agents. The latter factor is particularly important given the variable effects of anesthetic agents on CA; for instance, propofol and sevoflurane have been associated with the least impairment of *CA.* A combination of remifentanil and propofol induces a dose-dependent metabolism-coupled reduction in CBF with preserved CA making it ideal for cases where systemic hypotension is a concern (58, 59). In the General or Local anesthesia in intra-arterial therapy (GOLIATH) study which was a prospective randomized open label blinded end-point study, 128 subjects were randomized between GA and CS. It was found that while undergoing MT therapy for LVO, the GA group had significantly higher successful reperfusion compared to the CS arm. They also had a shift toward a lower mRS score at 90 days (60). The Sedation vs. Intubation for Endovascular stroke treatment (SIESTA) study showed no difference in NIHSS at 24 h which was the primary outcome, however there was a higher proportion of patients who had attained functional independence at 3 months in the GA arm compared with the CS arm (61). Given the discordance between earlier observational studies and more recent prospective randomized but single center open label studies, further RCT data is needed.

#### Effect of partial pressure of CO2(PaCO2) and end-tidal CO2

Hypocapnia (low PaCO2) causes vasoconstriction in the cerebral vasculature. Its effect on CA has yet to be completely understood. Minhas et al. studied the effects of end tidal carbon dioxide (ETCO2) on CA, CBF velocity (CBFv), autoregulatory index (ARI) and arterial blood pressure in 45 healthy volunteers and found a logistic curve relationship akin to a “dose response” curve for the effects of PaCO2 on the cerebral vasculature (62). Salinet et al. (11) demonstrated a lower CBFv step response to CO2 in the stroke group compared with controls suggesting a possible modulatory effect of hypocapnia on CA and neurovascular coupling (63). A single institution study in patients undergoing GA for MT demonstrated that end tidal carbon dioxide (ETCO2) at 60 and 90 min was observed to be significantly associated with the outcome; mean ETCO2 was higher in the good clinical outcome group at both 60 and 90 min (defined as 90-day mRS 0–3) (64). Interestingly, the difference in ETCO2 between the good and poor outcome groups was relatively small; 35.2 vs. 32.2 mmHg respectively, at 60 min and 34.9 vs. 31.9 mmHg respectively, at 90 min (64).

#### Effect of head or body position

The literature differs on the effect of head positioning on AIS outcome and *CA.* It has been shown that “lying flat” increases CBFV (65–67), CPP (68) and cerebral oxygenation (69) which in theory could improve perfusion in AIS. It has also been shown that there is a reduction in CA when patients with mild AIS go from “lying flat” to “sitting up” compared to controls without any significant changes in CBFV (70). A prospective randomized cluster study (HeadPoST) in patients with acute ischemic stroke did not show any differences in functional outcome between subjects who were “lying flat” or “sitting up” (71, 72). The median NIHSS was 4 in both arms likely suggesting that LVO patients did not constitute a significant proportion of the study population.

### Optimization of CA and clinical implications

**Figure 4:**
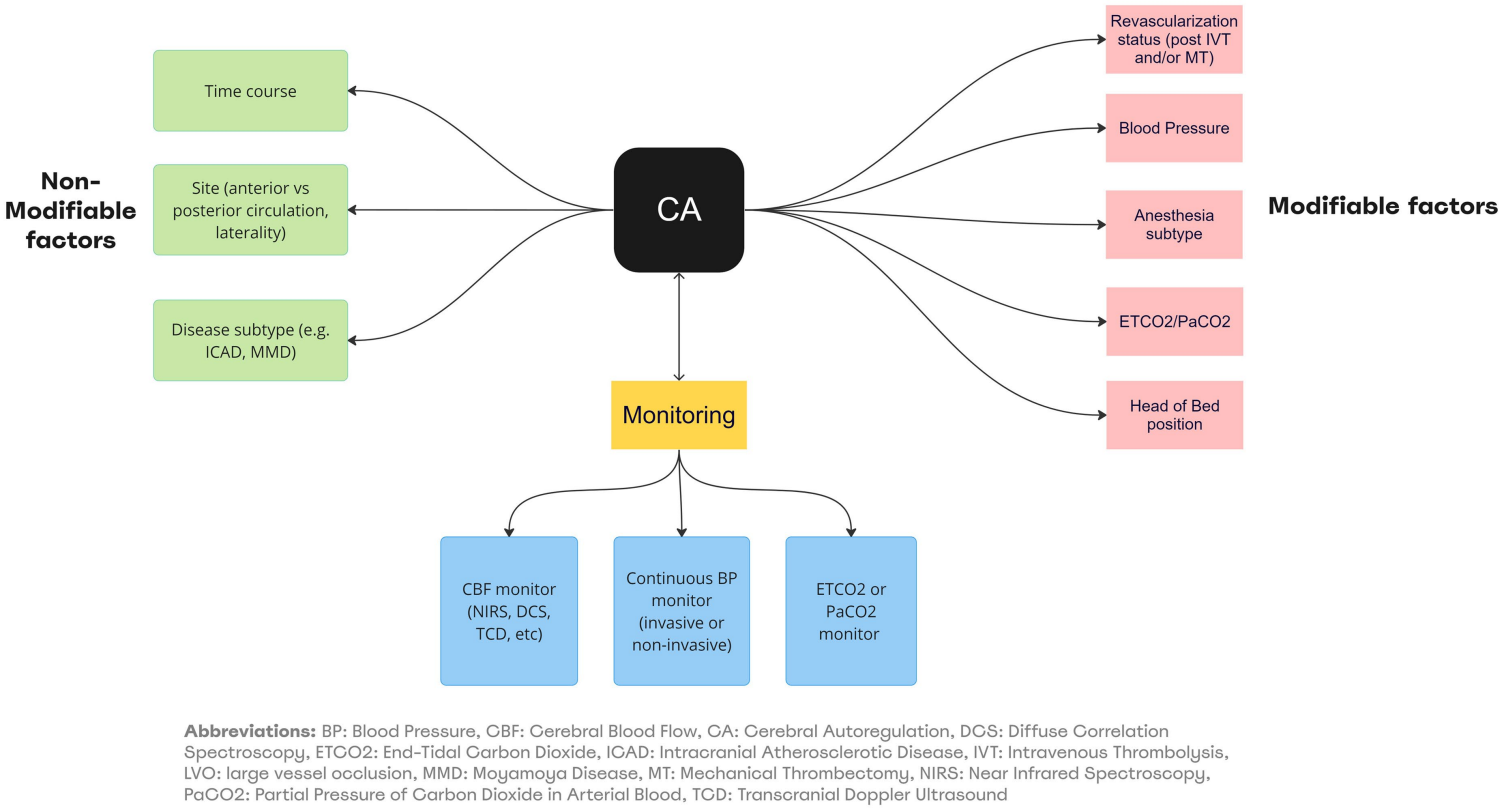
Summary of factors affecting CA/Neurovascular coupling and monitoring in LVO. BP, Blood Pressure; CBF, Cerebral Blood Flow; CA, Cerebral Autoregulation; DCS, Diffuse Correlation Spectroscopy; ETC02, End-Tidal Carbon Dioxide; ICAD, Intracranial Atherosclerotic Disease; IVT, Intravenous Thrombolysis; LVO, large vessel occlusion; MMO, Moyamoyo Disease; MT, Mechanical Thrombectomy; NIRS, Near-Infrared Spectroscopy; PaCO2, Partial Pressure of Carbon Dioxide in Arterial Blood; TCD, Transcranial Doppler Ultrasound.

In the absence of supportive data, guidelines on optimization of CA in clinical management of LVO patients are lacking. It is a common practice to adopt the following pragmatic approach which relies on an understanding of the interaction between the various physiologic factors affecting CA and neurovascular coupling (See Figure 1). This incorporates some evidence discussed in the previous sections, while awaiting prospective randomized clinical trials (See Table 2; Figure 4).

**Table 2:** Summary table of factors affecting CA and neurovascular coupling in LVO.

Non-modifiable factor(s)	Method of CA or CBF assessment (parameter)	Key findings	Author/Reference
Time	TFA (phase shift, gain, coherence)	CA impaired early and begins to normalize gradually at 72–96 h; CA impairment can persist beyond 7 days up to 3 months	Sheriff et al. (12) Kwan et al. (13)
Site a. Laterality	TFA (phase shift)	Worse CA in AH compared with UH Worse CA in hypo perfused viable tissue compared with normal tissue	Petersen et al. (14) Hecht et al. (15)
b. Posterior circulation	TFA (phase shift)	Basilar stenosis patients with decreased phase shift (worse CA) more likely to have new AIS and poor 90 day outcome	Gong et al., 2013 (24)
c. Anterior circulation (extracranial atherosclerosis)	Mx (correlation coefficient)	CA impaired not only in severe ICA (70–99%) but also moderate (>50%) stenosis	Tang et al. (30)
3. Specific disease subtype a. ICAD	TFA (phase, coherence) TFA (phase, gain) ARI	Ipsilateral CA in hypoperfusion related ICAD closely correlated to collateral status and CBF (MRI ASL) Asymptomatic MCA stenosis with ipsilateral CA impairment Symptomatic MCA stenosis with bilateral CA impairment	Tian et al. (32) Wang et al. (34) Gong et al. (36) Chen et al. (35)
b. MMD	TFA (RoR, phase, gain, coherence) NIRS, COx, HVx	Dynamic CA impaired even in early stages of MMD; autoregulatory parameters well correlated with angiographic stage of MMD Optimum MAP and LLA during indirect revascularization can be identified	Chen et al. (38) Lee et al. (39)

Modifiable factor(s)	Method of CA or CBF assessment (parameter)	Key findings	Author/Reference
Revascularization status	ARI TFA (phase shift, gain, coherence)	CA impaired in subjects not responding to IVT; had worse outcomes per early NIHSS Complete recanalization (TICI 3) associated with better CA profile than incomplete (TICI 2b or lower)	Nogueira et al. (42) Sheriff et al. (12)
BP	NIRS; time correlation analysis n/a n/a	MAP opt at which CA is most preserved can be calculated. More time within autoregulatory limits (AL) associated with less HT and infarct growth; more time above ULA associated with worse 90 day mRS Peak SBP post MT of 158 mm Hg able to discriminate better outcomes BP overcorrection (intensive SBP < 120 mmHg) post MT associated with worse outcomes	Petersen et al. (14) Mistry et al. (51) Yang et al. (50)
Anesthesia choice	n/a n/a n/a	Propofol and sevoflurane associated with less CA impairment Combination of remifentanil and propofol preserves CA (especially where concern for hypotension) No dedicated CA data comparing GA versus CS; Randomized trials SIESTA and GOLIATH showing trends in outcome benefit favoring GA but multicenter RCT pending	Messick et al. (58) Leslie-Mazwi et al. (59) Simonsen et al. (60)Schönenberger et al. (61)
PaC02/End tidal CO2	n/a	Intraprocedural MT ETCO2 higher in the good clinical outcome group (~35 compared with ~32 mm Hg)	Takahashi et al. (64)
Head-of-bed position	ARI n/a	Decrease in CA from lying to sitting position in mild AIS; HeadPoST study with median NIHSS 4 in general AIS showed no difference (No randomized data specific to LVO)	Lam et al. (70) Anderson et al. (71)

AH, Affected Hemisphere; AIS, Acute Ischemic Stroke; ARI, Autoregulation Index; ASL, Arterial Spin Labeling; BP, Blood Pressure; CA, Cerebral Autoregulation; CBF, Cerebral Blood Flow; CBFv, Cerebral Blood Flow Velocity; CMRO2, Cerebral Metabolic Rate of Oxygen Extraction; CVR, Cerebrovascular Resistance; CVRi, Cerebrovascular Resistance Index; Cox, Cerebral Oximetry Index; CPP, Cerebral Perfusion Pressure; CS, Conscious Sedation; CT, Computed Tomography; CrCP, Critical Closing Pressure; ETCO2, End-Tidal Carbon Dioxide; GA, General Anesthesia; HT, Hemorrhagic Transformation; HVx, Hemoglobin Volume Index; ICA, Intracerebral Atherosclerosis; ICAD, Intracerebral Atherosclerotic Disease; ICH, Intracerebral Hemorrhage; IVT, Intravenous Thrombolysis; LLO, Lower Limit of Autoregulation; LVO, Large Vessel Occlusion; MAPopt, Optimum Mean Arterial Pressure; MCA, Middle Cerebral Artery; MAP, Mean Arterial Pressure; MMD, Moya moya Disease; MRI, Magnetic Resonance Imaging; MT, Mechanical Thrombectomy; NIHSS, National Institutes of Health Stroke Scale; NIRS, Near Infrared Spectroscopy; PaCO2, Partial Pressure of Carbon Dioxide in Arterial Blood; PaO2, Partial Pressure of Oxygen; PET, Positron Emission Tomography; PI, Pulsatility Index; RCT, Randomized Control Trial; RoR, Rate of Recovery; SBP, Systolic Blood Pressure; TFA, Transfer Function Analysis; TICI, Thrombolysis in Cerebral Infarction; TNK, Tenecteplase; tPA, Tissue Plasminogen Activator; UH, Unaffected Hemisphere; ULA, Upper Limit of Autoregulation.

n/a, Not Applicable or Available (Refers to studies where CA was not an outcome variable).

Patients presenting with LVO in the pre-treatment phase are laid flat unless there is a concern for increased ICP or a high aspiration risk. An arterial line is placed prior to intervention and permissive systolic hypertension considering end-organ injury risk (up to 220 mmHg in non-thrombolysis eligible candidates and up to 180 mmHg in thrombolysis-eligible candidates) prior to and during intervention. Relative drops in systolic and mean arterial pressures prior to recanalization should be avoided. Upon attainment of revascularization, systolic blood pressures are lowered, usually to less than 160 mmHg and in some cases under 140 mmHg based on degree of recanalization and perceived hemorrhagic transformation risk. Tight systolic BP control under 120 mmHg is avoided except in cases with high risk for hyper perfusion syndrome such as in tandem strokes. These blood pressure parameters are observed for a minimum of 24 h and in certain cases for up to 96 h post recanalization. End tidal CO2 is commonly measured during and after treatment and a goal of ~35 mmHg is aimed for. Conscious sedation is performed in appropriate patients; in case general anesthesia is deemed necessary this decision is made early on. A dedicated anesthesiologist ensures maintenance of blood pressure and end tidal CO2 goals within the aforementioned parameters. In addition, either total intravenous anesthesia with propofol and/or remifentanil OR select inhalational gases such as sevoflurane are administered based on their favorable autoregulatory profiles. The ideal scenario will entail identifying optimum mean arterial pressure (MAP opt), limits of autoregulation (LA) and in certain cases optimum cerebral perfusion pressure (CPP opt) for each individual which will allow for real time adjustment of BP, end tidal CO2, ICP and even medication/ anesthestic agents. This precision based medicine strategy has the potential to improve clinical outcomes as suggested by preliminary work (14), but requires validation in larger cohorts of patients.

### Prognostication

#### Infarct growth

Intact CA during AIS has not only been shown to be responsible for a smaller infarct volume but also protects the vulnerable penumbra. Sudden drastic variations in blood pressure especially the diastolic component have been shown to cause penumbral loss (73). With timely revascularization, preserved CA within 6 h (phase >37 radians) is associated with smaller infarct volumes at 24 h (17). Regional assessment of CA using intraoperative laser speckle imaging showed reduced CA in the affected hemisphere with severe impairment of CA in the ischemic yet viable penumbra (15). An important caveat is that despite successful revascularization there can be penumbral tissue loss possibly due to reperfusion injury (74).

#### Hemorrhagic transformation

Hemorrhagic transformation (HT) is a serious complication of AIS yet its mechanism is still under debate. Various studies have shown that an early (<24 h) impairment of CA (decrease in phase) in AIS is a strong predictor of HT (14, 74). In a study focused on CA in LVO, early impaired CA was a predictor of type 2 parenchymal hematoma (PH2), which is usually associated with worsening in the neurologic exam (12).

## Conclusion

Cerebral autoregulation is impaired in LVO patients and leads to worse neurologic outcomes likely through secondary brain injury. In the presence of LVO, continuous CA monitoring may soon be a crucial tool for the periprocedural and intensive care management of these patients. A combination of multiple non-invasive modalities will likely provide a more accurate monitoring of both the cerebral macro- and micro-circulation(s) allowing a more comprehensive understanding of the actual state of CA. In unfavorable situations such as unsuccessful revascularization, absence of collateral flow or delayed treatment, tailored monitoring and management of CA may have an impact on the final outcome through a precision medicine based approach. Nonetheless, more research is needed to understand how impaired CA mediates ongoing brain injury. We need to identify associated physiological variables and the development of pharmacologic, neuroprotective or interventional therapies that could mitigate secondary brain injury with the ultimate goal of improving outcomes.

## Author contributions

FS: Conceptualization, Data curation, Formal Analysis, Methodology, Resources, Supervision, Writing – original draft, Writing – review & editing. AA: Data curation, Writing – original draft, Writing – review & editing. MI: Data curation, Formal Analysis, Investigation, Methodology, Software, Writing – review & editing. RK: Writing – review & editing. AM: Writing – review & editing. GR: Conceptualization, Data curation, Methodology, Supervision, Writing – original draft, Writing – review & editing.

## Glossary

ARI: Autoregulation index
ASL: Arterial Spin Labeling
BP: Blood Pressure
CBF: Cerebral Blood Flow
CBFv: Cerebral Blood Flow Velocity
CA: Cerebral Autoregulation
COx: Cerebral Oximetry Index
CPP: Cerebral Perfusion Pressure
CrCP: Critical Closing Pressure
CT: Computed Tomography
CVR: Cerebrovascular Resistance
CVRi: Cerebrovascular Resistance Index
ETCO2: End-Tidal Carbon Dioxide
GA: General Anesthesia
HT: Hemorrhagic Transformation
ICA: Internal Carotid Artery
ICAD: Intracranial Atherosclerotic Disease
ICH: Intracerebral Hemorrhage
ICP: Intracranial Pressure
IVT: Intravenous Thrombolysis
LLA: Lower Limit of Autoregulation
LVO: Large Vessel Occlusion
MCA: Middle Cerebral Artery
MAP: Mean Arterial Pressure
MMD: Moyamoya Disease
MRI: Magnetic Resonance Imaging
MT: Mechanical Thrombectomy
NIRS: Near Infrared Spectroscopy
NIHSS: National Institutes of Health Stroke Scale
PET: Positron Emission Tomography
PI: Pulsatility Index
RoR: Rate of Recovery
RCT: Randomized Control Trial
SBP: Systolic Blood Pressure
TFA: Transfer Function Analysis
TICI: Thrombolysis in Cerebral Infarction
AIS: Acute Ischemic Stroke
PaCO2: Partial Pressure of Carbon Dioxide in Arterial Blood
PaO2: Partial Pressure of Oxygen
HVx: Hemoglobin Volume Index
tPA: Tissue Plasminogen Activator
TNK: Tenecteplase
ULA: Upper Limit of Autoregulation
