# Supplementary material for: A systematic review on the assessment of cerebral autoregulation in patients with Large Vessel Occlusion
Source: Front Neurol. 2023 Nov 17;14:1287873. doi: 10.3389/fneur.2023.1287873 (PMC10693431; doi:10.3389/fneur.2023.1287873)
Supplement: Supplementary file 1 [file Data_Sheet_1.docx]

**APPENDIX I**

**Search Strategy**

PubMed (October 06, 2022)

- (((("Stroke"[Mesh]) OR ("Stroke*" OR "Cerebrovascular Accident*" OR "CVA (Cerebrovascular Accident)" OR "Cerebrovascular Apoplexy" OR "Brain Vascular Accident*" OR "Cerebrovascular Stroke*" OR "Apoplexy" OR "Cerebral Stroke*" OR "Acute Stroke*" OR "Acute Cerebrovascular Accident*" OR "acute ischemic stroke" OR "acute ischaemic stroke" OR ("large vessel occlusion*" OR "large-vessel intracranial occlusion*" OR "LVO"))) AND (((("Brain"[Mesh]) OR ("Encephalon")) OR (("Cerebrum"[Mesh]) OR ("Cerebra" OR "Right Cerebral Hemisphere" OR "Cerebral Hemisphere*" OR "Left Cerebral Hemisphere"))) OR (("Cerebrovascular Circulation"[Mesh]) OR ("Cerebral Circulation*" OR "Brain Blood Flow*" OR "Regional Cerebral Blood Flow" OR "Cerebral Perfusion Pressure*" OR "Cerebral Blood Flow*") OR ("cerebral")))) AND (("Homeostasis"[Mesh]) OR ("Autoregulation") OR ("dysautoregulation"))) AND ((((("Endovascular Procedures"[Mesh]) OR ("Endovascular Procedure*" OR "Intravascular Procedure*" OR "Intravascular Technique*" OR "Endovascular Technique*")) OR (("Thrombectomy"[Mesh]) OR ("Thrombectomies" OR "Percutaneous Aspiration Thrombectom*" OR "Aspiration Thrombectom*"))) OR (("Mechanical Thrombolysis"[Mesh]) OR ("Mechanical Clot Disruption*")) OR ("large vessel occlusion*" OR "large-vessel intracranial occlusion*" OR "LVO") OR (("Thrombolytic Therapy"[Mesh]) OR ("Therapeutic Thrombolys*" OR "Fibrinolytic Therap*" OR "Thrombolytic Therapies")) OR ("Tissue Plasminogen Activator"[Mesh]) OR ("Tissue Activator D-44" OR "Tissue Activator D 44" OR "Tisokinase" OR "Tissue-Type Plasminogen Activator" OR "Tissue Type Plasminogen Activator" OR "TTPA" OR "T-Plasminogen Activator" OR "T Plasminogen Activator" OR "Alteplase" OR "Activase" OR "Actilyse")))

EMBASE (October 11, 2022)

- ('cerebrovascular accident'/exp OR ('stroke*' OR 'cerebrovascular accident*' OR 'cva (cerebrovascular accident)' OR 'cerebrovascular apoplexy' OR 'brain vascular accident*' OR 'cerebrovascular stroke*' OR 'cerebrovascular strokes' OR 'apoplex*' OR 'cerebral stroke*' OR 'acute stroke*' OR 'acute cerebrovascular accident*' OR 'acute cerebrovascular lesion' OR 'acute focal cerebral vasculopathy' OR 'apoplectic stroke' OR 'brain accident' OR 'brain attack' OR 'brain blood flow disturbance' OR 'brain insult*' OR 'cerebral apoplexia' OR 'cerebral insult' OR 'cerebro vascular accident' OR 'cerebrovascular arrest' OR 'cerebrovascular failure' OR 'cerebrovascular injury' OR 'cerebrovascular insufficiency' OR 'cerebrovascular insult' OR 'cerebrum vascular accident' OR 'cryptogenic stroke' OR 'cva' OR 'insultus cerebralis' OR 'thrombotic stroke')) AND (('brain circulation'/exp OR ('cerebrovascular circulation' OR 'cerebral circulation*' OR 'brain blood flow*' OR 'regional cerebral blood flow' OR 'cerebral perfusion pressure*' OR 'cerebral blood flow*' OR 'cerebral')) OR ('brain'/exp OR ('cerebrum' OR 'encephalon' OR 'cerebra' OR 'right cerebral hemisphere' OR 'cerebral hemisphere*' OR 'left cerebral hemisphere'))) AND ('autoregulation'/exp OR 'homeostasis'/exp OR 'homeostasis and regulation'/exp OR 'dysautoregulation') AND (('endovascular surgery'/exp OR ('endovascular procedure*' OR 'intravascular procedure*' OR 'intravascular technique*' OR 'endovascular technique*')) OR ('thrombectomy'/exp OR ('thrombectomies' OR 'percutaneous aspiration thrombectom*' OR 'aspiration thrombectom*')) OR ('mechanical thrombectomy'/exp OR ('mechanical thrombolysis' OR 'mechanical clot disruption*' OR 'mechanical embolectomy' OR 'pharmacomechanical thrombectomy')) OR ('fibrinolytic therapy'/exp OR ('thrombolytic therapy' OR 'therapeutic thrombolys*' OR 'fibrinolytic therap*' OR 'thrombolytic therapies' OR 'fibrinolytic treatment' OR 'thrombolytic treatment')) OR ('tissue plasminogen activator'/exp OR ('tissue activator d-44' OR 'tissue activator d 44' OR 'tisokinase' OR 'tissue-type plasminogen activator' OR 'tissue type plasminogen activator' OR 'ttpa' OR 't-plasminogen activator' OR 't plasminogen activator' OR 'alteplase' OR 'activase' OR 'actilyse' OR 'ak 124' OR 'ak124' OR 'angiochinase' OR 'angiokinase' OR 'hapase' OR 'plasvata')) OR ('large vessel occlusion*' OR 'large-vessel intracranial occlusion*' OR 'lvo')) AND [humans]/lim AND [english]/lim
